# Societal images of Cannabis use: comparing three countries

**DOI:** 10.1186/1477-7517-9-21

**Published:** 2012-06-18

**Authors:** John A Cunningham, Jan Blomqvist, Anja Koski-Jännes, Kirsimarja Raitasalo

**Affiliations:** 1 The Centre for Addiction and Mental Health & University of Toronto, 33 Russell Street, Toronto, ON, M5S 2S1, Canada; 2 The Centre for Social Research on Alcohol and Drugs (SoRAD), Stockholm University, Stockholm, Sweden; 3 School of Social Sciences and Humanities, University of Tampere, Tampere, Finland; 4 National Institute of Health and Welfare, Helsinki, Finland

**Keywords:** Cannabis, Societal beliefs, Epidemiology

## Abstract

**Background:**

Differences in beliefs about Cannabis were compared between Canada, Sweden and Finland using nationally representative population surveys containing similar items.

**Findings:**

Compared to Finnish and Swedish respondents, Canadians were both more likely to have tried Cannabis and to view Cannabis as a less serious problem for society.

**Conclusions:**

These findings emphasize the extent to which views about Cannabis can vary. It is possible that views about Cannabis are, at least in part, a social construction influenced by media, social policy and exposure to the drug that varies from country to country.

## Background

Peoples’ beliefs about Cannabis appear to be related to the way the drug is presented in the media and treated by the legal system [1]. Comparing societal beliefs about Cannabis between different countries can reveal inconsistencies in the way that this drug is viewed. These comparisons have the potential to highlight how beliefs about a drug do not result solely from the experience of using Cannabis itself by illustrating how different societies can have different attitudes towards this same illicit substance. This paper will explore this issue using a recently collected set of general population surveys in Canada, Sweden, and Finland that were constructed to assess views on various addictions using a similar set of items.

## Methods

This study uses a cross-national data set to compare perceptions of addictions as a societal problem in Canada, Sweden, Finland and St. Petersburg, Russia [2,3]. Because the Russian survey did not ask questions about Cannabis use, findings for this substance were not reported as part of the previous country comparisons. The current analysis employed the random population surveys from Finland (n = 740), Sweden (n = 1098), and Canada (n = 864). Details of the surveys can be found elsewhere [2] but briefly, the Canadian survey was a random digit dialing survey with a response rate of 41% and the Finnish and Swedish surveys were mailed surveys with response rates of 37% and 55% respectively. Respondents on all surveys were asked a series of questions starting with an item that asked them to rate on a scale from 1 (not at all serious) to 10 (extremely serious), how serious they thought a number of different issues were to society (alcohol problems, property crimes, environmental damage, use of Cannabis, use of hard drugs, lacking gender equality, smoking, violent crimes, large wage differences, prostitution, poverty, gambling, ethnic discrimination, misuse of prescribed drugs, financial crimes; note that only results from Cannabis use are presented here). Next, respondents were asked how risky it was for someone to get addicted if they tried Cannabis even once (1 = low risk, 5 = high risk). The survey continued by asking if becoming addicted to Cannabis was the person’s fault or society’s, and whether dealing with the Cannabis addiction was the person’s responsibility or society’s. Next, the survey asked how likely a person was to be able to fix the Cannabis addiction on their own without treatment and then how likely a person would be to fix Cannabis addiction if they went to treatment (1 = no chance, 6 = very large chance). The survey concluded with a series of demographic questions. Proportions and statistical tests are presented as weighted values to be representative of the general population in the respective countries while sample sizes are reported as unweighted values. Statistical comparisons between countries were conducted using bivariate tests (Chi-square tests for catergorical variables and one-way ANOVA for the continuous variable) with a Bonferroni adjustment to account for multiple statistical comparisons (.05/5 test: *p* < .01).

## Results

Table 1 compares levels of Cannabis use and opinions about Cannabis in the three countries. There was substantial difference in levels of Cannabis use between the countries with Finland and Sweden having larger proportions of people who have never tried Cannabis (83.4% and 84.5%) as compared to Canada (58.9%; *p* < .001). In addition Canadians were less likely to believe that there was a very high risk of addiction to Cannabis if it was tried (11.4%) as compared to Finland (31.8%) and Sweden (27.1%; *p* < .001). Finally, there were differences in perceptions of Cannabis as a social problem with Canada rating Cannabis as a less serious problem, Finland taking a middle ground and Sweden the most serious (*p* < .001). In addition, when the rank ordering of the different problems asked about were compared between countries, there were substantial differences in the ordering observed. Canada ranked Cannabis as the least serious of 15 societal problems while Finland ranked it in the middle (7^th^ out of 15) and Sweden as one of the more serious of societal problems (5^th^ out of 15).

**Table 1:** Comparing Cannabis use and opinions between three countries

	**Finland (n = 740)**	**Sweden (n = 1098)**	**Canada (n = 864)**	***p***
Cannabis use				
% past 12 months	4.4	1.7	11.9	
% prior to past 12 months	12.2	13.8	29.2	
% never	83.4	84.5	58.9	.001
Mean (SD) seriousness of Cannabis use as a societal problem ^a^	6.6 (2.6)	8.2 (2.3)	5.6 (2.6)	.001
% Very high risk of addiction if try	31.8	27.1	11.4	.001
% Large chance of fixing addiction on own, without treatment	10.4	7.8	7.9	N.S.
% Large chance of fixing addiction with treatment	21.4	25.6	20.4	N.S.

N.S. = not significant*, p* > .01.

^a^ 1 = not at all serious; 10 = extremely serious.

## Discussion

Cannabis is perhaps the best example of a drug for which societal attitudes could be expected to vary. This is because it lies at the cusp between illegal and legal in some countries. In Canada, for example, attitudes toward Cannabis have been becoming steadily more favorable over the past decades [4]. Further, the use of Cannabis for medical purposes is now legal in Canada and there have been ongoing initiatives to decriminalize this drug [4]. In countries such as Sweden and Finland, use of Cannabis is still a criminal offence with no special consideration given for use of the drug for medical purposes. In addition, there has been a long history of demonizing Cannabis as a serious drug of abuse [5]. How do these variations regarding the legal nature of Cannabis use correspond with societal attitudes regarding the Cannabis use?

The three countries in this cross-national survey displayed quite different attitudes towards Cannabis. Canada viewed Cannabis as having minimal risk, both as a problem for society as well as for the person as a drug that is unlikely to result in addiction if it is tried. Participants in Sweden and Finland were considerably more concerned about Cannabis as a problem in their society, with Sweden displaying the most concern and Finland scoring somewhere between Sweden and Canada. Interestingly, these differences appear to mirror actual levels of Cannabis use in each country where more than 40% of Canadian report having tried Cannabis at least once while less than 20% of people in Sweden and Finland report ever trying Cannabis.

The congruence between levels of actual Cannabis use and beliefs about the seriousness of Cannabis as a problem also lends weight to the assertion made by Blomqvist [6] that drugs which people are familiar with are viewed as of relatively low risk while unfamiliar drugs are viewed as having high risk for the individual and society. In the current analysis, the observation is played out at the country level as Cannabis is a drug that Canadians seem quite familiar with and they regard it as of relatively little concern while people in Sweden and Finland are relatively unfamiliar with Cannabis and they view this drug as fairly dangerous. The differences in beliefs about Cannabis between the three countries also highlight the extent to which our views about this illicit drug may in part be a social construction, reflecting beliefs promulgated in the media as well as policies and laws regarding the drug [1,4,5]. Otherwise, how could the same drug be viewed as dangerous in one country and relatively benign in another?

## Competing interests

The authors declare that they have no competing interests.

## Authors’ contributions

JAC, JB, and AK conceived of the project, constructed the survey items, and ran the surveys in each of the respective countries. KR merged the data files. JAC conducted the analyses and wrote-up the paper. All authors contributed to and have approved the final manuscript.
